# Human Skin Model From 15 GHz to 110 GHz

**DOI:** 10.1002/bem.70025

**Published:** 2025-10-04

**Authors:** Andreas Christ, Adrian Aeschbacher, Bernadetta Tarigan, Ninad Chitnis, Arya Fallahi, Sven Kühn, Myles Capstick, Niels Kuster

**Affiliations:** ^1^ Foundation for Research on Information Technologies in Society (IT'IS) Zurich Zurich Switzerland; ^2^ Schmid and Partner Engineering AG Zurich Zurich Switzerland; ^3^ Tarigan Statistical Consulting Dübendorf Zurich Switzerland; ^4^ Department of Information Technology and Electrical Engineering Swiss Federal Institute of Technology (ETH) Zurich Zurich Switzerland

**Keywords:** 5G NR FR2, dielectric tissue properties, dosimetry, millimeter wave exposure

## Abstract

Compliance testing of wireless devices with absorbed power density (APD) limits requires body models that conservatively reproduce the absorption characteristics of human skin. Previous studies indicate that impedance‐matching effects are caused by the stratum corneum (SC) layer. The objective of this study is to develop a single macroscopic dielectric model reproducing absorption of electromagnetic fields by the skin up to 110 GHz. The reflection coefficient of the skin of human volunteers was measured at frequencies of 15 to 43 GHz with open waveguide probes, complementing previous data from 45 to 110 GHz. The measurements were made at various regions of the body. The statistical analysis of the results shows that the reflection coefficient in dB follows normal distribution in regions with thin SC, which permits the development of a conservative skin model. In regions with thick SC, for example, the palms, the reflection coefficient is not normally distributed because the thickness of the SC depends on the mechanical stress the hands are exposed to. The measured data allow the derivation of dispersive two‐layer models representing absorption and reflection at the skin surface with known uncertainty. The models can be used to conservatively demonstrate compliance with the APD limits of wireless devices in any of the 5G and 6G bands.

## Summary

- Broadband evaluation of the skin reflection coefficient with open waveguide probes.
- Corroboration of increased absorption of millimeter wave radiation in body regions with increased stratum corneum thickness.
- Dispersive dielectric models representing reflection and absorption at the skin surface with known population coverage.

## 1 Introduction

In the most recent revisions of the exposure safety guidelines, the International Commission on Nonionizing Radiation Protection (ICNIRP) (ICNIRP 2020) and the IEEE International Committee on Electromagnetic Safety (IEEE ICES) (IEEE C95.1. 2019) introduce basic restrictions based on absorbed power density (APD) (ICNIRP 2020) and epithelial power density (IEEE C95.1. 2019) averaged over an area of 4 cm^2^ for frequencies of 6–300 GHz, and 1 cm^2^ for frequencies above 30 GHz at the surface of the body. The objective of these restrictions is to limit the maximum macroscopic temperature increase at the surface of the skin. It is argued that the APD is an appropriate quantity to achieve this objective, because, at these frequencies, the penetration depth is small, and the incident electromagnetic (EM) power is predominantly absorbed in the surface layers (Foster et al. 1998; Alekseev and Ziskin 2009).

Testing compliance of wireless devices in terms of the APD requires the use of phantoms that can adequately simulate reflection and penetration of EM fields by human skin and allow the APD to be determined with known uncertainty (IEC/IEEE DTR 63572. 2025; Chitnis et al. 2025). Similar requirements exist for phantoms used to test over‐the‐air (OTA) performance of wireless devices (CTIA Test plan for mobile station over the air performance, Revision 3.9 2019). The development of such phantoms is based on the frequency‐dependent reflection and penetration parameters for any polarization of incident fields.

The most widely used dielectric parameters for skin tissues (Baumgartner et al. 2024) are derived from open coaxial probe (OCP) measurements of the human forearm skin (Gabriel et al. 1996a; Gabriel et al. 1996b; Baumgartner et al. 2024) at frequencies of up to 20 GHz. The OCP method provides accurate values for homogeneous materials, but has serious limitations when used to measure layered structures like the skin. A recent review of the dielectric properties of biological tissues (Sasaki et al. 2022) includes discussions of coaxial probes (Chahat et al. 2011; Zhekov et al. 2019), time‐domain spectroscopy (Pickwell et al. 2004; Sasaki et al. 2017), and open‐ended waveguide probes (Alekseev and Ziskin 2007). Time‐domain spectroscopy can be used only on excised tissue samples in vitro, whereas OCPs and waveguide probes can be applied for in vivo measurements. Open‐ended waveguide probes measure the reflection coefficient of the fundamental transverse electric (TE_10_) mode and have already been applied to skin tissue (Alekseev and Ziskin 2007; Alekseev et al. 2008; Christ et al. 2021), whose reflection characteristics were characterized with the help of simple stratified models for frequencies up to 100 GHz.

More detailed models were proposed for microdosimetry of the skin. These include, for example, the sweat glands or the ruggedness of its layers (Feldman et al. 2009; Betzalel et al. 2018). However, planar stratified models have proven to be sufficient for the quantification of macroscopic absorption of electromagnetic radiation. For this purpose, the safety guidelines (IEEE C95.1. 2019; ICNIRP 2020) define basic restrictions in terms of the spatial‐average absorbed power density.

The envelope of minimum and maximum absorption of electromagnetic radiation in the frequency range from 6 to 100 GHz in the skin was evaluated by means of a stratified model of tissue layers of variable thicknesses (Christ et al. 2020), which demonstrated increased absorption of millimeter waves at frequencies greater than 20 GHz due to impedance matching effects in the stratum corneum (SC) layer. However, measurements of the skin of 37 volunteers made in different regions of the body confirm that the difference in the thickness of the SC of the palm compared to the other parts of the body results in considerable differences in reflection and transmission (Christ et al. 2021).

## 2 Objectives

The findings reported by (Christ et al. 2021) were limited to the frequency range of 40 to 110 GHz. The objective of that work was to develop phantoms for evaluations of the OTA performance of handheld and body‐mounted devices operated at frequencies above 6 GHz. However, the findings were inadequate for the derivation of skin models to represent the user group with conservative coverage (typically > 90%) for demonstrating compliance with the APD exposure limits in the near‐field of handheld and body‐worn wireless devices (ICNIRP 2020; IEEE C95.1. 2019).

The goals of this study are to

- Extend the work of (Christ et al. 2021) to close the frequency gap between 15 GHz and 45 GHz;
- quantify reflection and absorption of human skin within regions with thin and thick SC;
- develop simple stratified dielectric models for the evaluation of the macroscopic absorption characteristics of the skin;
- consider different user‐group coverage factors with respect to the conservativeness of the exposure assessment.

In addition, the validity and accuracy of the applied measurement method were checked with the help of a homogeneous gel phantom with known dielectric parameters covered with low‐permittivity layers of Teflon (polytetrafluoroethylene [PTFE]).

## 3 Materials and Methods

### 3.1 Instrumentation

**Figure 1:**
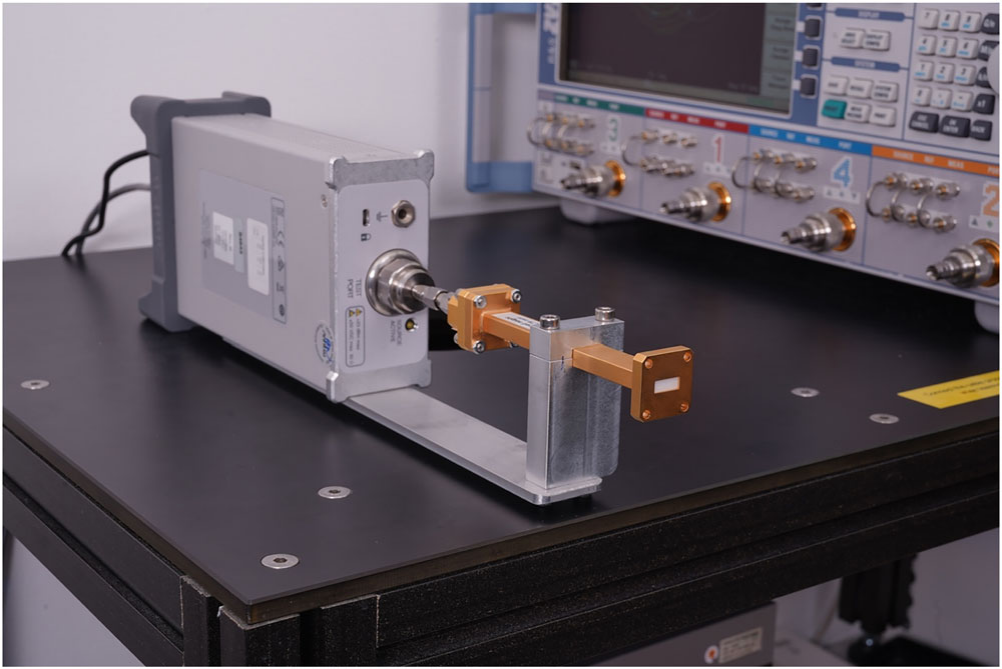
Measurement setup with the WR42 waveguide capped with rigid low permittivity foam and fixed with an aluminum bracket.

WR42 and a WR28 open‐ended rectangular waveguides with flanges from Eravant (Torrence CA, USA) were connected to an Anritsu (Atsugi, Kanagawa, Japan) model MS46131A vector network analyzer (VNA). The WR42 waveguide was used to analyze the 14–28 GHz frequency band, and the WR28 was used to analyze the 21–42 GHz band. Figure 1 shows the measurement setup with the WR42 waveguide.

To ensure that the surface of the soft tissues, such as skin, is flat across the flange openings, pieces of rigid high‐density low permittivity foam of 4.8 mm thickness were inserted into the flange openings. The dielectric parameters of the foam insert from Rohacell HF51 (Darmstadt, Germany) were measured over the of 5–67 GHz frequency range with the DAK‐1.2E‐TL2 dielectric probe kit for thin‐layer materials from Schmid & Partner Engineering AG (Zurich, Switzerland). In addition, the measured data were compared to measurements made with the DAK‐R resonator from Schmid & Partner Engineering AG at discrete frequencies of 10, 17, 26, 35, and 45 GHz determined as $\epsilon_{r}=1.09\pm 0.03$ and $\tan\delta =2.68\times 10^{-3}\pm 3\times 10^{-4}$ (Schmid & Partner Engineering AG. 2025). To ensure mechanical stability during the measurements, the waveguides were fixed in a customized bracket.

The WR42 and WR28 measurement setups were calibrated over their respective frequency ranges with a short, a λ/4 offset short, and with the STQ‐TO‐42‐S1‐CKIT1 and STQ‐TO‐28‐S1‐CKIT loads from Eravant. The low‐loss foam and the adapter to connect the waveguide to the coaxial feed‐line were included in the calibration. The reference plane for S_11_ is the interface between the waveguide flange and the measured sample.

### 3.2 Simulation of the Measurement Setup

The measurement setup described in Section 3.1 was modeled computationally with the help of mode matching (MM) based on a code developed specifically for rectangular waveguides that are terminated with a stratified dielectric. The code calculates the reflection coefficient of the TE_10_‐mode under the assumption that the waveguide flange is infinitely large and takes the fringing fields at the waveguide opening into consideration. As was observed in (Christ et al. 2021), the impact of the fringing fields on the reflection coefficient is relevant and must be taken into account, in particular for low permittivity dielectrics, such as the SC layer. A detailed description of the MM implementation for open waveguides can be found in (Chitnis et al. 2025). The implementation was validated with the help of measurements of a well‐characterized coated dielectric as well as of simulations performed with the finite‐difference time‐domain (FDTD) method (Section 4).

### 3.3 Volunteer Study

**Figure 2:**
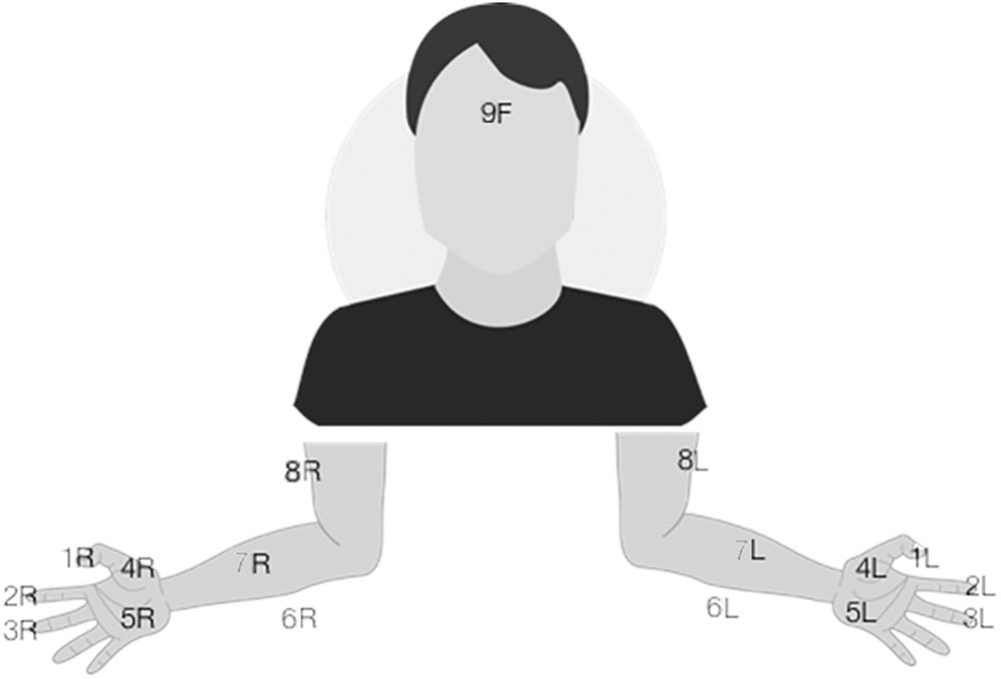
Anatomical sites on the hands, arms, and faces of the volunteers where S_11_ was measured; F denotes the forehead, L the left side, and R the right side.

Table 1 provides an overview of the age groups and sexes of the volunteers, and the S_11_ measurement sites on the hands, arms, and face (Figure 2) with thick versus thin SC are listed in Table 2. The assignment of the measurements sites in Figure 2 to skin with thick and thin SC layer had already been confirmed in (Christ et al. 2021). Manual labor and hobbies that involve physical or mechanical stress and friction on the hands are expected to be associated with an above‐average increase of the thickness of the SC on the palms and fingers. The professions of 11 of the adult volunteers (over 20 years of age) in the study require manual labor; 25 are employed as office workers.

**Table 1:** Age and sex distribution of the volunteer participants. Data for WR15 and WR10 taken from (Christ et al. 2021).

Age group	Age (years)	Sex	Number of subjects	
			WR42, WR28	WR15, WR10
Child	5–10	Female	—	2
		Male	—	2
Child	10–15	Female	3	—
		Male	5	—
Adult	20–40	Female	6	5
		Male	6	8
Adult	41–60	Female	7	7
		Male	8	8
Elderly	61–80	Female	3	2
		Male	6	3
Total			44	37

**Table 2:** Measurement locations defined in Figure 2 with thin versus thick SC layers.

	Regions
Thin SC	1F, 6R, 6L, 7L, 7R, 8L, 8R
Thick SC	1L, 1R, 2L, 2R, 3L, 3R, 4L, 4R, 5L, 5R

The measurements of the reflection coefficients S_11_ of the volunteers were carried out according to the protocol applied in the earlier study (Christ et al. 2021):

- The reflection coefficients of the skin of the 44 volunteers were measured at the anatomical sites shown in Figure 2.
- For each measurement, the waveguide was pressed against the skin of the volunteer. The distance between the waveguide flange and the measured surface, corresponding to the reference plane used for calibration, is considered to be zero.
- Three consecutive S_11_ measurements were taken, and the mean value was calculated.^1^
- Measured values of all regions with thick versus thin SC (as listed in Table 2) were grouped and averaged.
- Mean and standard deviation values^2^ for the two regions and the four waveguides (WR42, WR28, WR15, and WR10)^3^ were calculated, and the summary statistics for the different volunteer groups were evaluated.

**Figure 3:**
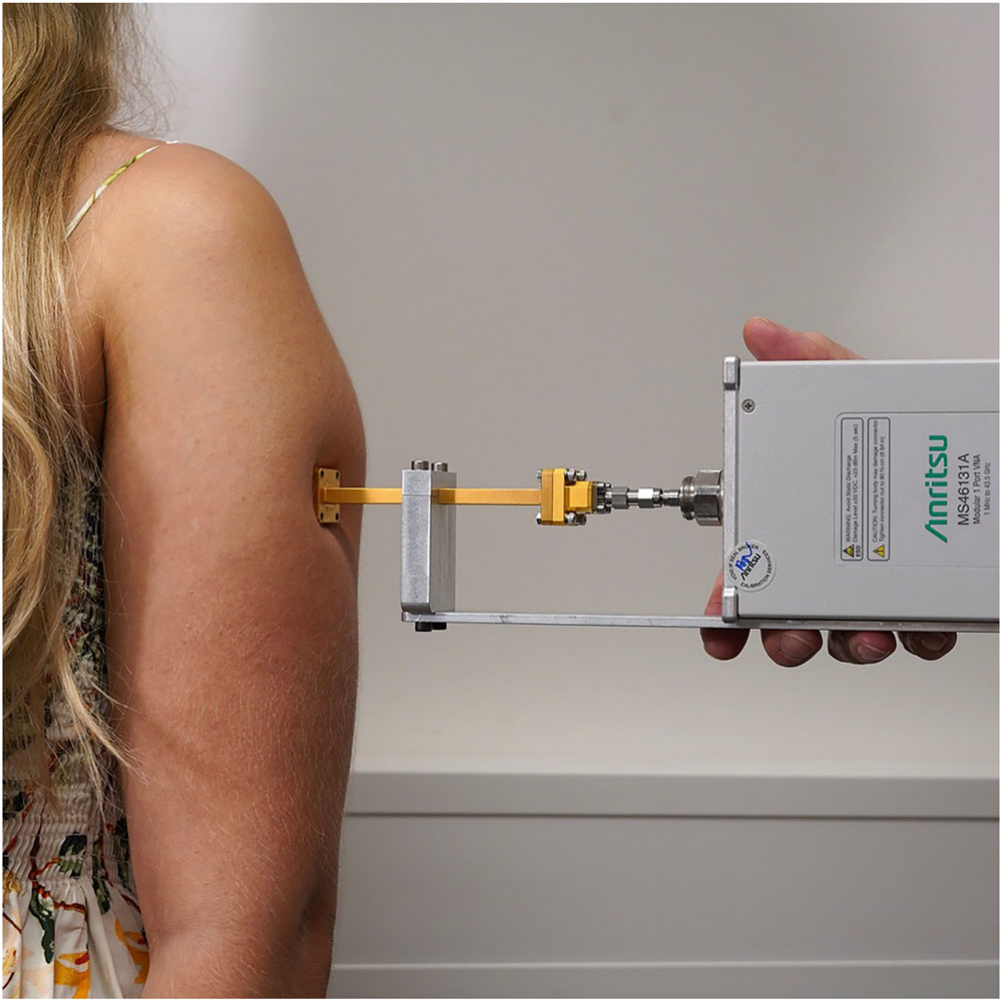
Measurement of the reflection coefficient from a volunteer at skin site 8L.

The volunteers were instructed about purpose of the study and about the single steps of the experimental procedure. Informed consent was obtained by all volunteers, or in case of the children, by their legal representatives. Figure 3 shows a reference measurement of a volunteer.

### 3.4 Skin Model and Statistical Analysis

As was reported in (Christ et al. 2021), absorption and reflection of the complex skin can be well approximated with a two‐layer model stratified as a surface layer–referred to as Layer SC–and an inner layer–referred to as Layer D. This study applies the same approach to determine the thicknesses of the layers and to derive coefficients for broadband models that describe their dielectric parameters according to the volunteer measurements (Section 3.3).

The dielectric parameters of the material models of the skin (Section 5.3) and of the gel used for the validation measurements (Section 4) are represented by dispersive Cole–Cole and Debye models. The complex permittivity of the Cole–Cole model is given as

(1) $$\epsilon_{r_{CC}}=\epsilon_{r_{\infty }}+\frac{\epsilon_{r_{s}}-\epsilon_{r_{\infty }}}{1+{\left(j\omega \tau \right)}^{1-\alpha }}-\frac{j\sigma }{\epsilon_{0}\omega },$$

where $\epsilon_{r_{\infty }}$ is the relative permittivity of the material when the frequency approaches infinity, $\epsilon_{r_{s}}$ is static relative permittivity, σ is the static conductivity, τ is the relaxation time, and α is a parameter of value between 0 and 1. For α=0, Equation (1) corresponds to the Debye dispersion model.

The parameters of the dielectric model of Equation (1) and the thickness of the Layer SC were fitted to the reflection coefficients of the volunteer measurements by means of sequential quadratic programming (SQP) in the implementation of GNU Octave (Eaton et al. 2022). For the fitting, the waveguide reflection coefficients of the skin model were calculated with MM (Section 3.4). Finally, plane wave reflection coefficients were calculated analytically for the skin models on the basis of the fitted dispersion models. For the statistical analysis, R (version 4.0.3) and RStudio (version 1.3.1093) (R Core Team 2017; RStudio Team 2020) were applied.

## 4 Validation of the Applied Methods

The waveguide probe method (Section 3.1) and the calculation of the reflection coefficient by MM (Section 3.4) were validated with a homogeneous lossy gel covered with PTFE with thicknesses of 50 µm and 100 µm. The dielectric properties are determined over a frequency range from 5 GHz to 67 GHz with the dielectric probe kit DAK 1.2E of Schmid & Partner Engineering AG. The uncertainty of the DAK 1.2E for this evaluation is < 0.2 dB.

The reflection coefficient of the tissue‐simulating gel was measured with the waveguides WR42, WR28, WR15, and WR10. The numerical models of these waveguides, including their flanges and terminating with a layered dielectric were simulated by means of the FDTD method in Sim4Life Version 8.4 (Sim4Life.science, Zurich MedTech AG, Zurich, Switzerland). The implementation was verified according to (IEC/IEEE 62704‐1 2017).

**Figure 4:**
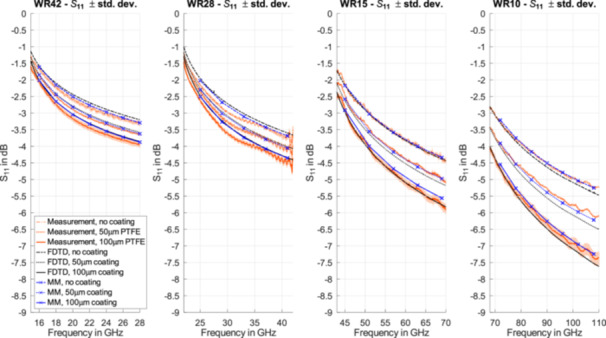
Measured reflection coefficients (mean of three measurements ± 1 standard deviation [shading]) of the TE_10_ mode of the tissue‐simulating gel coated with PTFE layers with thicknesseses of 50 µm and 100 µm in front of the four waveguides used to cover the different frequency regions, compared to MM and FDTD simulations performed according to the dispersive layer model based on the parameters summarized in Table 3.

The dielectric parameters of the gel can be represented by a Cole–Cole model as summarized in Table 3. The numerical results for the WR10 waveguide were obtained by extrapolation of the Cole–Cole model of the gel to frequencies up to 110 GHz. For the FDTD simulations, separate Debye models were fitted to this Cole–Cole model for the frequency ranges of each waveguide, and the waveguides were modeled with their flanges. Figure 4 shows the measured reflection coefficient compared to the results obtained with the MM and FDTD simulations. For all results, the maximum deviation is less than 0.5 dB.

**Table 3:** Dielectric models (Equation [1]) of the layered material used to validate the open waveguide probe setup.

	d	$\epsilon_{r_{\infty }}$	$\epsilon_{r_{s}}$	σ	τ	α
	mm			S/m	ps	
Gel	> 20	4.62	42.5	1.02	11.8	0.075
PTFE	0.05 and 0.1	2.1	2.1	0	0	0

## 5 Results

### 5.1 Statistical Evaluation of the SC Thickness

The distribution of S_11_ was tested for log‐normal distribution, that is, normal distribution in dB, at five distinct frequencies of the measurements with the WR42 and the WR28 waveguides. Table 4 summarizes the results of the Shapiro‐Wilk test for regions with thick and thin SC (as indicated in Figure 2). A log‐normal distribution could be demonstrated for both waveguides at all frequencies for thin SC only (*p* > 0.05). The measurement results for S_11_ in locations with thick SC show a 2‐ to 3‐fold increase in variance, which was expected because SC thickness is a function of mechanical stress and friction of the palm and the fingers and is therefore not log‐normally distributed. This is illustrated in Figure 6, which shows several outliers that have S_11_ values of up to 0.5 dB lower than what would be expected for a log‐normal distribution.

**Table 4:** The Shapiro‐Wilk tests on the difference of S_11_ (in dB) between thin and thick SC.

Waveguide	Frequency	SC	p
WR42	17.5 GHz	Thin	0.21
		Thick	< 0.01
	20 GHz	Thin	0.19
		Thick	< 0.01
	22.5 GHz	Thin	0.75
		Thick	< 0.01
	25 GHz	Thin	0.99
		Thick	< 0.01
	27.5 GHz	Thin	0.73
		Thick	< 0.01
WR28	24 GHz	Thin	0.61
		Thick	< 0.01
	28 GHz	Thin	0.33
		Thick	< 0.01
	32 GHz	Thin	0.16
		Thick	< 0.01
	36 GHz	Thin	0.40
		Thick	< 0.01
	40 GHz	Thin	0.45
		Thick	< 0.01

This result differs from the findings reported by (Christ et al. 2021), where log‐normal distribution could be shown for the difference between S_11_ at all frequencies of 60 GHz and above, with only one exception at 45 GHz. The different outcome can be explained by the more homogeneous group of volunteers in the earlier study.

**Figure 5:**
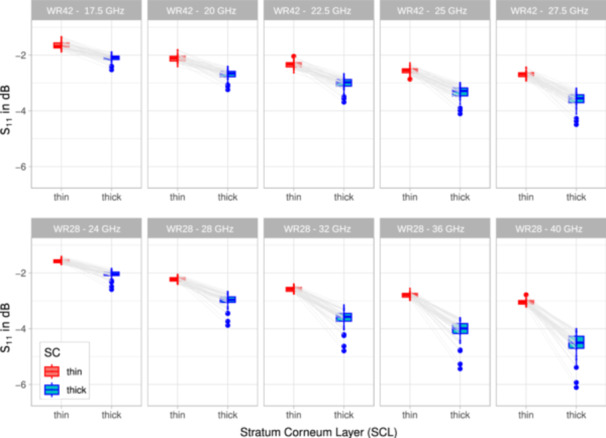
Paired box plots and paired t‐tests (*p* < 2.2 × 10^−16^) for the S_11_ measurements performed at skin sites with thin (red) versus thick SC (blue) layers in all 44 volunteers using the WR28 (top row) versus the WR42 (bottom row) waveguide.

While normal distributions were assumed for body regions with thin SC only in the evaluations described in Sections 5.3 and 5.2 of this study, two‐sided paired *t*‐tests were carried out for the differences between S_11_ measurements of thick and thin SC, yielding a *p*‐value of less than $2.2\times 10^{-16}$, which justifies the conclusion that the reflection coefficient of skin tissue can be classified as two distinct groups corresponding to thin and thick SC. Paired box plots and paired *t*‐tests of the S_11_ measurements of thick and thin SC are shown in Figure 5 and Table 5.

**Table 5:** Results of the two‐side paired *t*‐tests on the difference in reflection coefficient between thin SC and thick SC $\Delta S_{11}$ and the lower and upper limits of the 95th percentile confidence interval $l_{l}$ and $l_{u}$; *p* is always smaller than 2.2 × 10^−16^.

Waveguide	Frequency	Mean $\Delta S_{11}$	$l_{l}$	$l_{u}$
WR42	17.5 GHz	0.47	0.43	0.52
	20 GHz	0.58	0.52	0.64
	22.5 GHz	0.69	0.62	0.76
	25 GHz	0.79	0.71	0.87
	27.5 GHz	0.91	0.82	1.00
WR28	24 GHz	0.49	0.44	0.54
	28 GHz	0.77	0.69	0.85
	32 GHz	1.05	0.95	1.16
	36 GHz	1.27	1.15	1.40
	40 GHz	1.53	1.40	1.67

### 5.2 Statistics of the Volunteer Groups

In addition to the analysis of the SC thickness (Section 5.1), possible differences due to volunteer sex and age were evaluated. Weak evidence for sex‐dependent differences had been observed previously in (Christ et al. 2021). The two‐sample *t*‐tests carried out on the measurement data in this study yielded *p*‐values much larger than 0.05 (> 0.5 in most cases), that is, there was no confirmation of any evidence for sex‐dependent differences. The Bartlett tests indicate equal variances (*p* ≥ 0.30).

To test for age dependence, only the groups of young adults (20–40 years) and the elderly (60–80 years) were compared. An age dependent decrease of the power reflection in the order of magnitude of 0.5 dB at frequencies of 26 GHz and 60 GHz had been reported in (Sacco et al. 2021) based on a numerical evaluation of the assumed effects of age dependent physiological changes on the dielectric properties of tissue. For the comparison of the data of this study, the frequency dependence of the mean S_11_ was removed by normalizing the individual S_11_ to the respective mean value for each frequency. The two‐sample *t*‐tests indicate that the *p*‐values are much smaller than 0.05, which suggests that age has an effect on the reflection coefficient of skin with thin SC. The observed decrease in the reflection coefficient for the elderly volunteers with respect to the young adult volunteers, however, is < 0.2 dB. This tendency is regarded as too small for consideration in the development of the dielectric skin models (Section 5.3), but may confirm the results of (Sacco et al. 2021). Again, the Bartlett tests indicate equal variances (*p* ≥ 0.13).

### 5.3 Dielectric Skin Models for Different Coverage Factors

The mean values and standard deviations of the reflection coefficients measured with the four waveguides are shown in Figure 7. The data for the WR15 and the WR10 waveguides were taken from (Christ et al. 2021). As already observed in Figure 5 and discussed in Section 5.1, the distributions of the reflection coefficients of thin and thick SC are clearly different. A Debye model for Layer D was fitted to the mean value of the reflection coefficient. For the fit, Layer SC with the dielectric parameters of (Ziskin et al. 2018) was used. The reported thicknesses in the literature range from less than 10 µm (Holbrook and Odland 1974; Talreja et al. 2001) to 20 µm and above (White et al. 1987; Egawa et al. 2007). Its electrical thickness is in the order of magnitude of λ/100 at 100 GHz. Its impact on the overall reflection coefficient is regarded as small, and it is therefore not used as a free parameter for the SCP fit of the properties of the skin model (Section 3.4). For this study, a fixed value of 20 µm is used.

The results of the fit are shown in Figure 7, and the Debye parameters for Layer D are given in Table 6. Deviations of the fit from the measurement results are less than 0.1 dB. For a more conservative exposure estimate, additional models were fitted to the mean value of S_11_ minus one and two standard deviations. These correspond to the 68th and 95th percentiles of the coverage of the reflection coefficient and can therefore be used to assess the APD of locations with thin SC with known population coverage.

**Table 6:** Parameters of the Debye models (Equation [1] with α=0) of the optimized dielectric models for layer thickness d. The parameters of Layer SC thin are taken from (Ziskin et al. 2018).

	Comment	dμm	$\epsilon_{r_{\infty }}$	$\epsilon_{r_{s}}$	σ S/m	τ ps
Layer D	Mean S 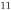	∞	7.88	47.0	5.19	8.35
	68% coverage	∞	5.06	37.0	7.62	8.19
	95% coverage	∞	2.98	32.8	6.94	8.76
Layer SC	Thin	20	2.96	4.46	0	6.9
	Thick 1	227	4.01	9.11	0	1.63
	Thick 2	262	3.27	8.22	0	2.04
	Thick 3	295	2.49	7.29	0	2.22

For the reflection coefficient of skin with thick SC, an alternative dispersive model for Layer SC, thick 1, was developed, for which a value of 227 µm for the thickness was obtained from the fitting process. This value lies within the order of magnitude of the upper limit of the SC thickness reported, for example, in (Fruhstorfer et al. 2000; Welzel et al. 2004; Egawa et al. 2007) for the palm and the fingers. Additional measurement results for Layer SC, thick 2 and thick 3, were fitted for the 68th and the 95th percentiles of the distribution of the measured reflection coefficient for thick SC.

The plane wave reflection coefficients of the skin models and for the dry skin model of (Gabriel et al. 1996b) with Layer SC of 20 µm thickness were calculated analytically and are shown in Figure 8. For the 95th percentile skin model with thin SC, the reflection coefficient ranges from − 2.9 dB at 15 GHz to − 6.0 dB at 110 GHz. The difference between the mean value and the more conservative 95th percentile model ranges from 0.3 dB at 15 GHz to 1.0 dB at 110 GHz.

This model represents conservative skin absorption, whereas the model of (Christ et al. 2021), developed for OTA applications, reflects average values of the hands, the feet, and the body at frequencies >45 GHz derived from the same data set, and offers comparable performance to existing phantoms below 6 GHz.

## 6 Discussion and Conclusions

In this study, we investigated the reflection characteristics of human skin with the help of open waveguide measurements performed on 44 volunteers at defined locations with thin and thick SC, covering a frequency range of 15–43 GHz. The results confirm earlier observations–from a previous study for frequencies between 40 GHz and 110 GHz–(Christ et al. 2021) that absorption of EM energy is increased in regions with thick SC. The measurement results of both studies are sufficiently similar and could therefore be combined for evaluation. In detail, the findings can be summarized as follows:

- In the frequency range of 15 to 43 GHz, the difference in S_11_ could be shown to be statistically significant for the palms and the inner side of the fingers in comparison to the remaining body regions, which confirms the findings of previous studies.
- No evidence could be found for the dependence of the S_11_ for body regions with thin SC on the sex of the volunteers.
- When the youngest adult group (20–40 years) is compared to the elderly (60–80 years), we observed a slight increase–less than 0.2 dB–in absorption of EM energy as a function of age.
- The combined results could be used to derive skin models to represent different coverage factors of the reflection and absorption characteristics for thin SC.
- Additional models for thick SC could also be derived as a function of the thickness of the SC layer.
- The plane wave reflection coefficient of skin with thin SC ranges from −2.9 dB at 15 GHz to −6.0 dB at 110 GHz for the 95th percentile of the population of the study.

**Figure 6:**
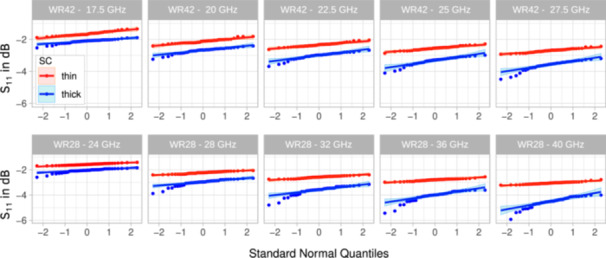
QQ plots of the S_11_ measurements in all 44 volunteers using the WR42 (top row) and the WR28 (bottom row) waveguides at the locations depicted in Figure 2 for thin (red) versus thick SC (blue).

The study has the following limitations:

- The number of participants was too small to reliably detect subtle differences in reflection and absorption coefficients between demographic groups (sex, age, ethnicity, etc.). Nevertheless, the results for thin SC, as discussed in Section 5.2, show small standard deviations (reflected in narrow 95% confidence intervals), and each of the paired *t*‐tests in Table 5 yields a *p*‐value of < 2.2 × 10^−16^. With the exception of lower reflection coefficients measured in the hands of volunteers doing manual work, Figure 6 reveals no outliers in our measurements despite the heterogeneity of the sampling pool of 44 volunteers. This consistency supports the conclusion that the measured reflection and absorption results are broadly valid.

- The study does not provide any information on the extent to which physical stress alters reflection/absorption.
- The developed skin model is a macroscopic model which is intended for the development of body phantoms to test compliance with safety limits. It does not incorporate the microstructure of the skin and is therefore not suitable for microdosimetry applications.

Based on the extended frequency range covering all currently used 5G bands, a dielectric model for skin tissue is proposed that can be applied for the conservative assessment of the APD with a population coverage of ≈ 95%.

**Figure 7:**
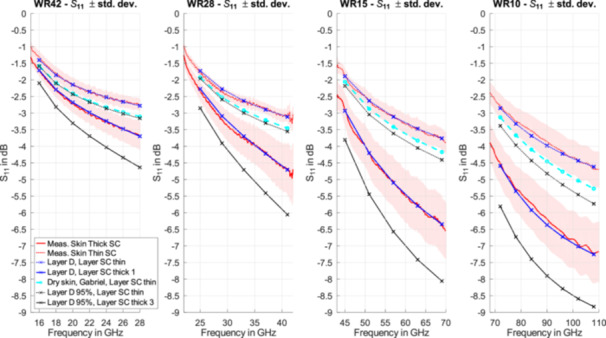
Reflection coefficients (mean of three measurements ±1 standard deviation [shading]) of the TE_10_‐mode using waveguides covering the different frequencies at locations with thin and thick SC and the optimized Debye models for Layer D and Layer SC. For comparison, the reflection coefficients of the dermis model of (Gabriel et al. 1996b)–evaluated with Layer SC–are also shown.

**Figure 8:**
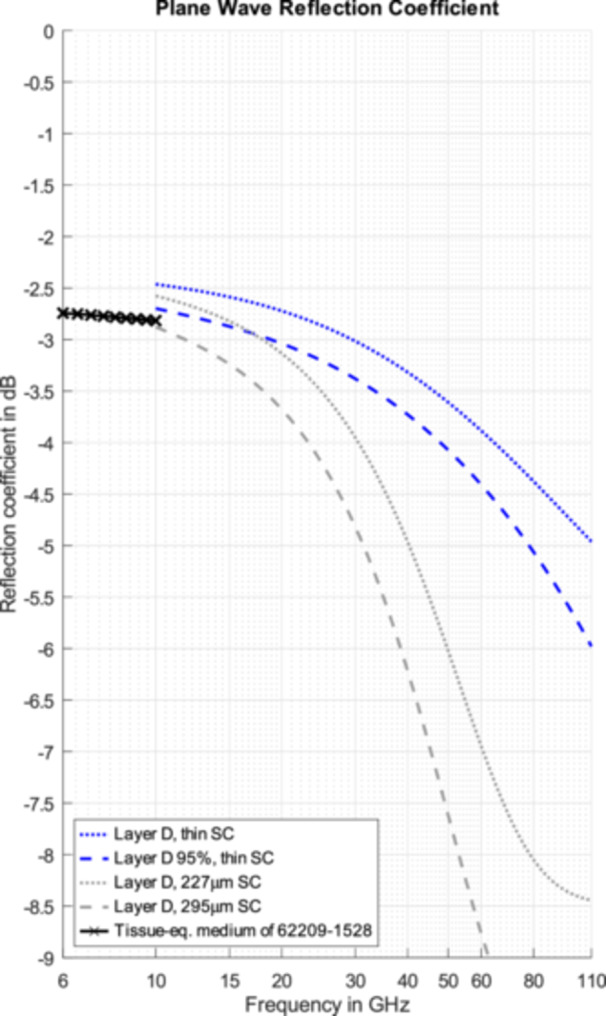
Plane wave reflection coefficients of the proposed conservative skin models for compliance testing of APD for a frequency range extending from 10 GHz to 110 GHz and compared to the tissue‐equvalent medium (between 6 and 10 GHz) specified in (IEC/IEEE 62209‐1528 2020).

## Conflicts of Interest

Niels Kuster is the founder of Schmid & Partner Engineering AG (SPEAG) and ZMT Zurich MedTech AG (ZMT). He is also a minority shareholder of NFT Holding AG that owns shares of SPEAG and ZMT. Adrian Aeschbacher is employed by SPEAG, which develops phantoms for over‐the‐air performance testing and provides measurement equipment and simulation tools for assessing the power density of wireless devices. ZMT develops simulation tools and numerical anatomical phantoms. Andreas Christ is a consultant of the Mobile and Wireless Forum.

## Data Availability

The authors have nothing to report.

## Endnotes

1 In total, 28 measurements showing discontinuities in the slope of S_11_ were considered outliers and excluded from further processing.

2 See the discussion of the statistical distribution of the measurement results in Section 5.1.

3 The data for WR15 and WR10 were taken from (Christ et al. 2021).
